# Supplementary material for: Tumoricidal, Temozolomide- and Radiation-Sensitizing Effects of KCa3.1 K+ Channel Targeting In Vitro Are Dependent on Glioma Cell Line and Stem Cell Fraction
Source: Cancers (Basel). 2022 Dec 15;14(24):6199. doi: 10.3390/cancers14246199 (PMC9776522; doi:10.3390/cancers14246199)
Supplement: Supplementary file 1 [file cancers-14-06199-s001.zip › cancers-1953331-supplementary.pdf]

Tumoricidal, Temozolomide- and Radiation-Sensitizing Effects of $K_{Ca}3.1$ K^+ Channel Targeting In Vitro Are Dependent on Glioma Cell Line and Stem Cell Fraction

Nicolai Stransky [1,2], Katrin Ganser [1], Ulrike Naumann [3], Stephan M. Huber [1,*] and Peter Ruth [2]

[1] Department of Radiation Oncology, University of Tübingen, 72076 Tübingen, Germany
[2] Department of Pharmacology, Toxicology and Clinical Pharmacy, Institute of Pharmacy, University of Tübingen, 72076 Tübingen, Germany
[3] Molecular Neurooncology, Hertie Institute for Clinical Brain Research and Center Neurology, University of Tübingen, 72076 Tübingen, Germany
* Correspondence: stephan.huber@med.uni-tuebingen.de or stephan.huber@uni-tuebingen.de; Tel.: +49-7071-29-82183; Fax: +49-7071-29-4944

Supplementary Figures

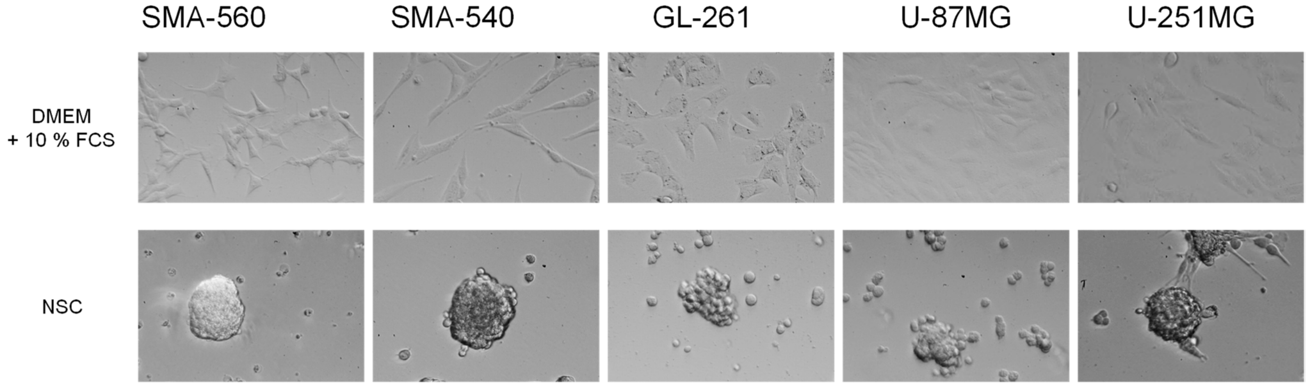

Supplementary Figure S1. Representative images of growth phenotypes in "bulk" cell-"differentiating" DMEM/10% FBS (top) and glioma stem cell-enriching NSC (bottom) medium. Note the adherent and spheroide-like phenotype of DMEM-and NSC-grown cells, respectively.

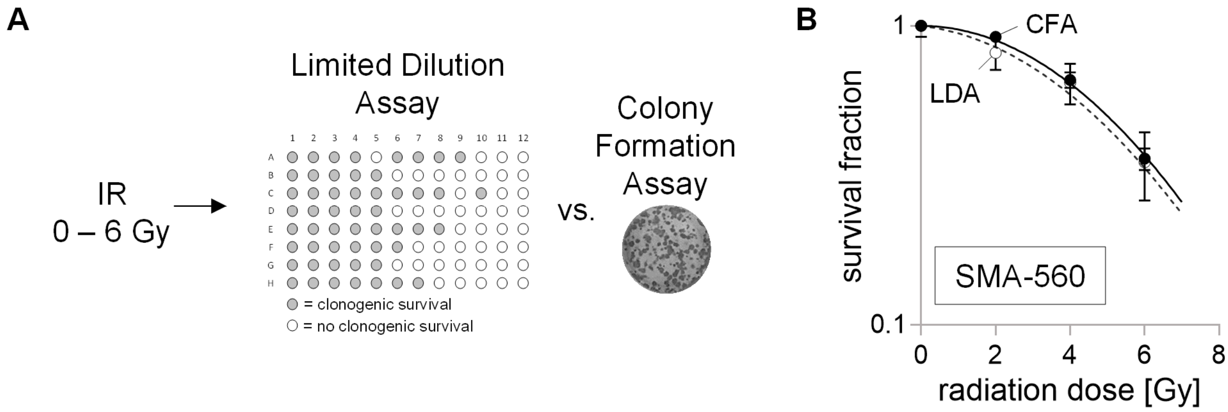

Supplementary Figure S2. Survival fractions of irradiated (0-6 Gy) SMA-560 cells grown in DMEM + 10% FBS determined by limited dilution assay (LDA; B, open circles) or colony formation assay (CFA; B, closed circles) are not different. Depicted is in (A, left) a schematic drawing of a 96-well plate where SMA-560 cell suspensions were sequentially 1:2-diluted (well 1 to 12 from left to right). Seeded cell number-dependent clonogenic growth after 7 d incubation is indicated by grey color. In (A, right) a micrograph of fixed and stained SMA-560 colonies typically obtained in colony formation assay is shown. Data in (B) are mean values (± standard error) of three experimental units and three to four observational units each. Survival fractions were fitted according to the linear quadratic model and given as follows: CFA, solid line; LDA, dashed line.

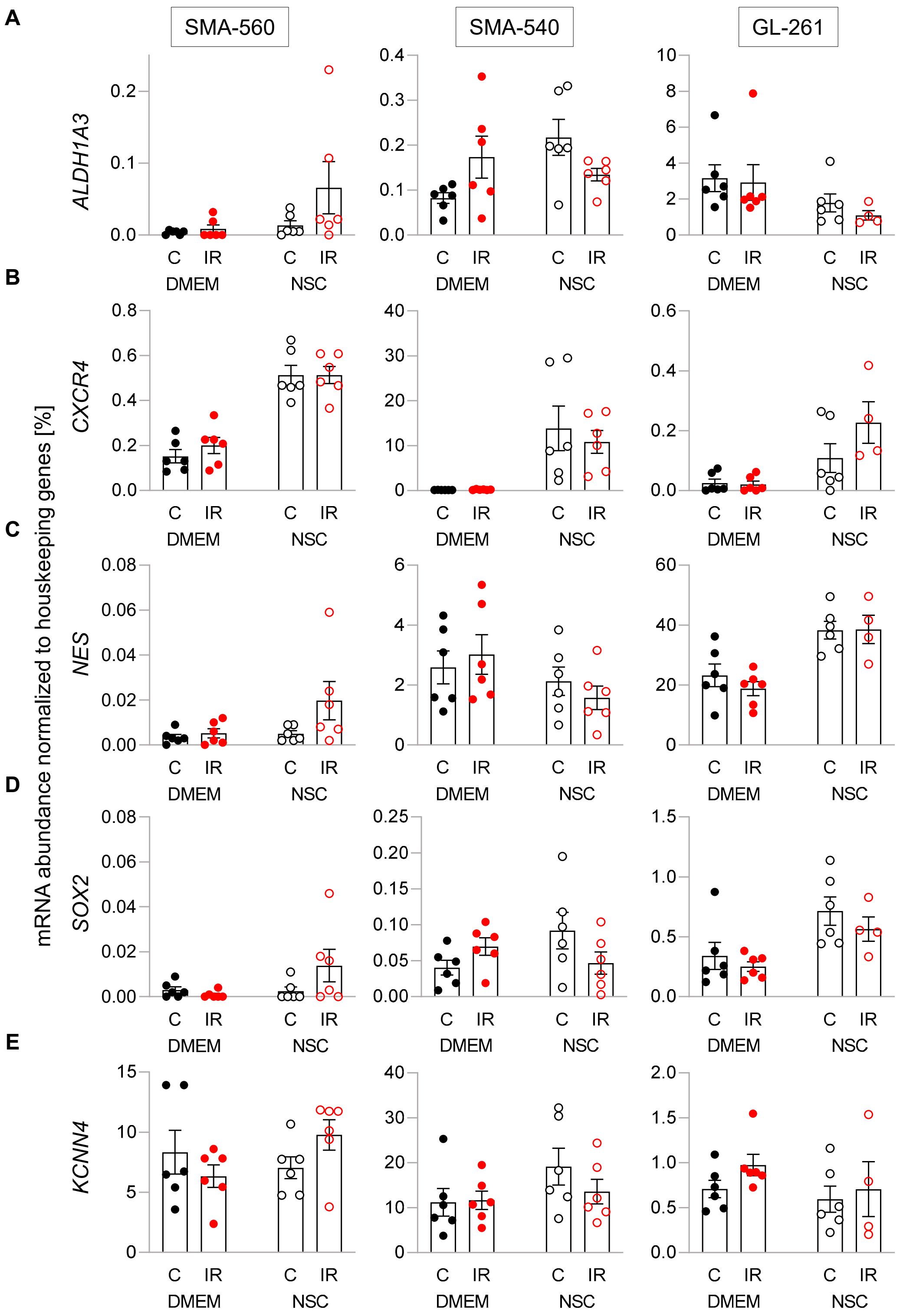

Supplementary Figure S3. Irradiation does not affect mRNA abundance of (A) *ALDH1A3*, (B) *CXCR4*, (C) *Nestin*, (D) *SOX2* or (E) *KCNN4* in SMA-560 (left), SMA-540 (middle) or GL-261 (right) cells systematically irrespective of culture condition. Cells were irradiated with 4 Gy and incubated for another 24 hours before cell pellets were collected and subsequently analyzed via RT qPCR. Bar graphs indicate mean values (± standard errors) of two to three experimental units and two observational units each. C = control (0 Gy), IR = irradiation with 4 Gy.

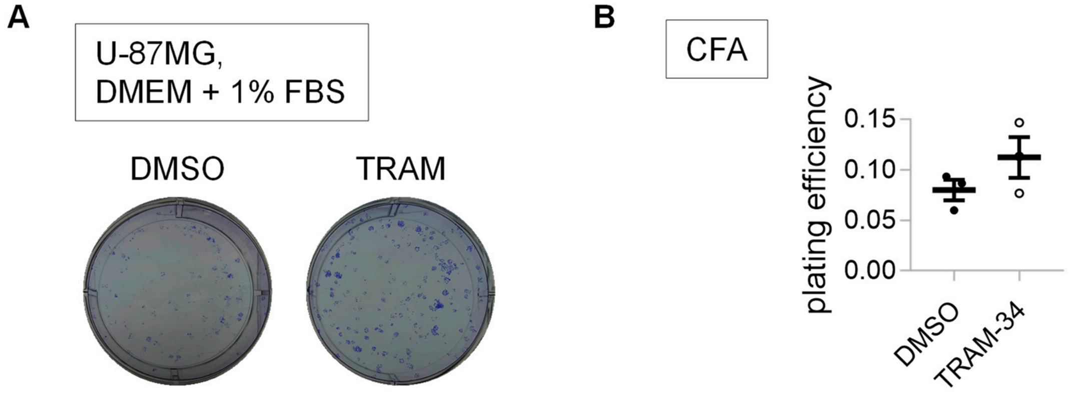

Supplementary Figure S4. TRAM-34 does not exhibit tumoricidal effects on U-87MG cells when incubated in DMEM + 1% FBS. A-B. Representative images of colony formation (A) and plating efficiencies (B) after treatment with vehicle (DMSO, closed black circles) or TRAM-34 (5 µM, open circles). Data are individual values and mean ± standard error of one experimental unit with three observational units each.
